# 2-Chloro­quinazolin-4(3*H*)-one

**DOI:** 10.1107/S1600536812022891

**Published:** 2012-05-31

**Authors:** Dong-Lei Cao, Fan-Yong Yan, Meng Wang, Chu-Ying Li, Li Chen

**Affiliations:** a School of Materials Science and Engineering, Tianjin Polytechnic University, Tianjin 300160, People’s Republic of China; b School of Environment & Chemical Engineering, Tianjin Polytechnic University, Tianjin 300160, People’s Republic of China

## Abstract

In the title compound, C_8_H_5_ClN_2_O, the quinazoline system is approximately planar with a maximum deviation from the least-squares plane of 0.034 (2) Å. In the crystal, classical N—H⋯O and weak non-classical C—H⋯N hydrogen bonds link the mol­ecules.

## Related literature

For the synthesis, see: Feng *et al.* (2007 ▶). For applications of related compounds, see: Labuda *et al.* (2009 ▶).
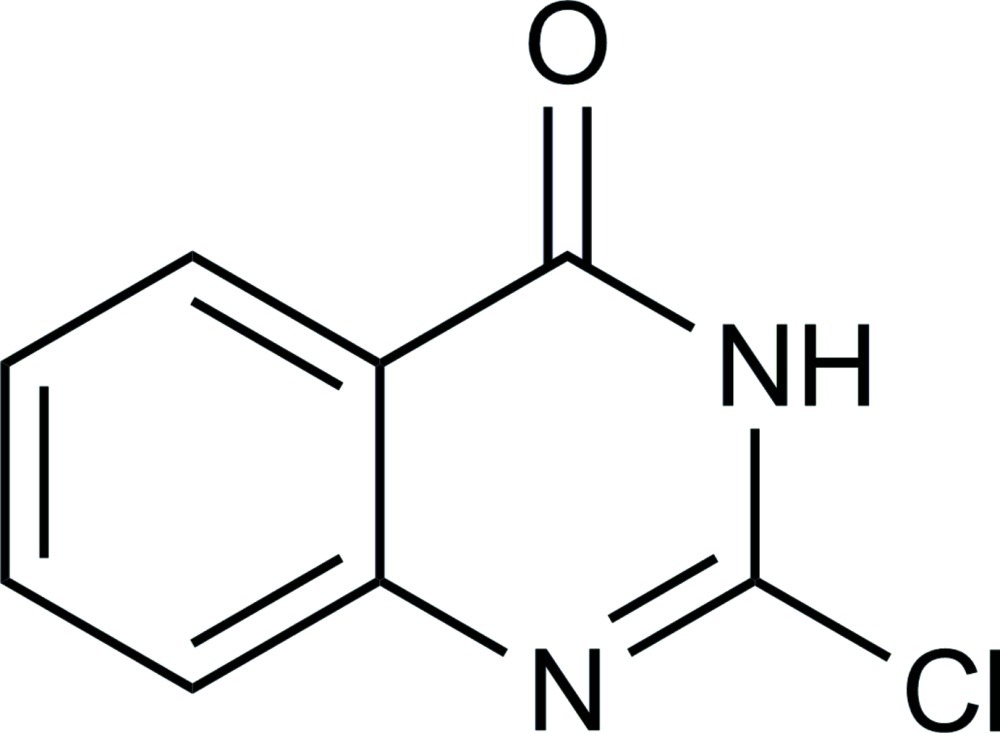


## Experimental

### 

#### Crystal data

C_8_H_5_ClN_2_O
*M* *_r_* = 180.59
Monoclinic, 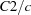

*a* = 22.4315 (16) Å
*b* = 3.7666 (6) Å
*c* = 18.0640 (13) Å
β = 104.682 (7)°
*V* = 1476.4 (3) Å^3^
*Z* = 8
Mo *K*α radiation
μ = 0.46 mm^−1^
*T* = 113 K
0.20 × 0.18 × 0.14 mm

#### Data collection

Rigaku Saturn CCD diffractometer
Absorption correction: multi-scan (*CrystalClear*; Rigaku/MSC, 2005 ▶) *T*_min_ = 0.914, *T*_max_ = 0.939
6933 measured reflections
1749 independent reflections
1430 reflections with *I* > 2σ(*I*)
*R*_int_ = 0.038

#### Refinement

*R*[*F* ^2^ > 2σ(*F* ^2^)] = 0.036
*wR*(*F* ^2^) = 0.095
*S* = 1.03
1749 reflections
113 parameters
H atoms treated by a mixture of independent and constrained refinement
Δρ_max_ = 0.45 e Å^−3^
Δρ_min_ = −0.23 e Å^−3^

### 

Data collection: *CrystalClear* (Rigaku/MSC, 2005 ▶); cell refinement: *CrystalClear*; data reduction: *CrystalClear*; program(s) used to solve structure: *SHELXS97* (Sheldrick, 2008 ▶); program(s) used to refine structure: *SHELXL97* (Sheldrick, 2008 ▶); molecular graphics: *SHELXTL* (Sheldrick, 2008 ▶); software used to prepare material for publication: *CrystalStructure* (Rigaku/MSC, 2006 ▶).

## Supplementary Material

Crystal structure: contains datablock(s) I, global. DOI: 10.1107/S1600536812022891/rk2353sup1.cif

Structure factors: contains datablock(s) I. DOI: 10.1107/S1600536812022891/rk2353Isup2.hkl

Supplementary material file. DOI: 10.1107/S1600536812022891/rk2353Isup3.cml

Additional supplementary materials:  crystallographic information; 3D view; checkCIF report

## Figures and Tables

**Table 1:** Hydrogen-bond geometry (Å, °)

*D*—H⋯*A*	*D*—H	H⋯*A*	*D*⋯*A*	*D*—H⋯*A*
N2—H1⋯O1^i^	0.92 (2)	1.88 (2)	2.7840 (17)	166.5 (19)
C3—H3⋯N1^ii^	0.95	2.53	3.449 (2)	163

Symmetry codes: (i) 
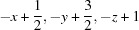
; (ii) 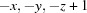.

**Fig. 1.**
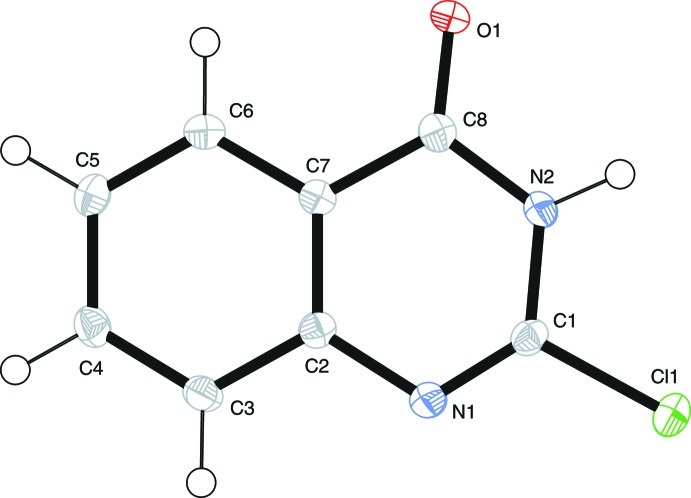
The molecular structure of title compound with the atom numbering scheme. Displacement ellipsoids are drawn with 50% probability level. H atoms are presented as spheres of arbitrary radius.

**Fig. 2.**
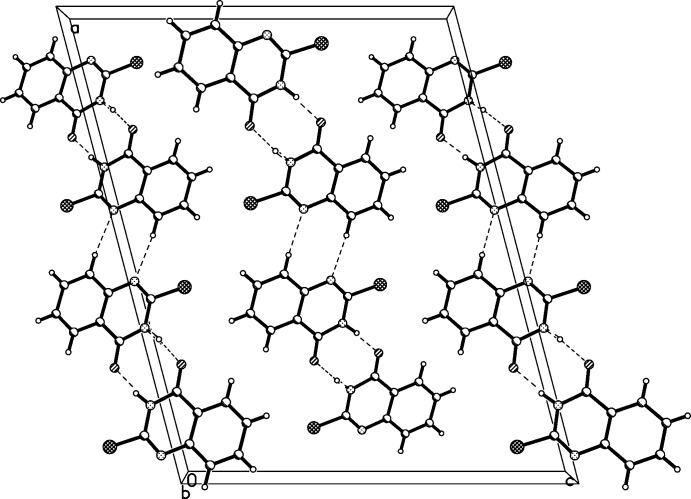
A packing diagram for title compound. Dashed lines indicate hydrogen bonds.

## supplementary crystallographic information

### Comment

Quinazolin-4(3*H*)-ones and related quinazolines are classes of fused heterocycles that are of consider able interest because of the diverse range of their biological properties, for example, anticancer, anti-inflammatory, diuretic, anticonvulsant and antihypertensive activities (Labuda *et al.*, 2009). The title compound (Fig. 1) consists of a quinazoline ring with an Cl atom at C4. The quinazoline heterobicycle is nearly planar, with a maximum deviation from the least-squares plane of 0.034 (2)Å. All bond lengths and angles are normal, atoms O1 and Cl lie in the 2-chloroquinazolin ring (C1-C8/N1/N2) plane. In addition, two intermolecular hydrogen bonding (classical N–H···O and non-classical C–H···N) (Table 1, Fig. 2), are effective in stabilizing the crystal structure.

### Experimental

The title compound was prepared by following a reported procedure (Feng *et al.*, 2007). A suspension of 2,4-dichloro-quinazolines (2.0 g, 1 mmol) was stirred in 2% aqueous sodium hydroxide solution (3 ml) for 3 h. Reaction mixture was diluted with water (6 ml) and filtered to remove unreacted 2,4-dichloroquinazoline. Filtrate was neutralized with dilute acetic acid, precipitate thus obtained was filtered and washed with water. The product was recrystallized from acetone / ethyl acetate (5:1) over 5 d at ambient temperature, gave colourless single crystals of 2-chloroquinazolin-4(3*H*)-one, suitable for X-ray analysis.

### Refinement

The H atoms based on C atoms were positioned geometrically at distances C–H = 0.93Å and constrained to ride on their parent atoms with *U*_iso_(H) = 1.2*U*_eq_(C). The amino H atom was found from different Fourier map and refined isotropically.

### Crystal data

**Table:**

C_8_H_5_ClN_2_O	*F*(000) = 736
*M**_r_* = 180.59	*D*_x_ = 1.625 Mg m^−^^3^
Monoclinic, *C*2/*c*	Mo *K*α radiation, λ = 0.71073 Å
Hall symbol: -C 2yc	Cell parameters from 2442 reflections
*a* = 22.4315 (16) Å	θ = 1.9–27.9°
*b* = 3.7666 (6) Å	µ = 0.46 mm^−^^1^
*c* = 18.0640 (13) Å	*T* = 113 K
β = 104.682 (7)°	Prism, colourless
*V* = 1476.4 (3) Å^3^	0.20 × 0.18 × 0.14 mm
*Z* = 8	

### Data collection

**Table:**

Rigaku Saturn CCD diffractometer	1749 independent reflections
Radiation source: rotating anode	1430 reflections with *I* > 2σ(*I*)
Multilayer monochromator	*R*_int_ = 0.038
Detector resolution: 14.63 pixels mm^-1^	θ_max_ = 27.9°, θ_min_ = 1.9°
ω and φ scans	*h* = −28→27
Absorption correction: multi-scan (*CrystalClear*; Rigaku/MSC, 2005)	*k* = −4→4
*T*_min_ = 0.914, *T*_max_ = 0.939	*l* = −23→23
6933 measured reflections	

### Refinement

**Table:**

Refinement on *F*^2^	Primary atom site location: structure-invariant direct methods
Least-squares matrix: full	Secondary atom site location: difference Fourier map
*R*[*F*^2^ > 2σ(*F*^2^)] = 0.036	Hydrogen site location: inferred from neighbouring sites
*wR*(*F*^2^) = 0.095	H atoms treated by a mixture of independent and constrained refinement
*S* = 1.03	*w* = 1/[σ^2^(*F*_o_^2^) + (0.0578*P*)^2^] where *P* = (*F*_o_^2^ + 2*F*_c_^2^)/3
1749 reflections	(Δ/σ)_max_ < 0.001
113 parameters	Δρ_max_ = 0.45 e Å^−^^3^
0 restraints	Δρ_min_ = −0.23 e Å^−^^3^

### Special details

**Table:**

Geometry. All s.u.'s (except the s.u. in the dihedral angle between two l.s. planes) are estimated using the full covariance matrix. The cell s.u.'s are taken into account individually in the estimation of s.u.'s in distances, angles and torsion angles; correlations between s.u.'s in cell parameters are only used when they are defined by crystal symmetry. An approximate (isotropic) treatment of cell s.u.'s is used for estimating s.u.'s involving l.s. planes.
Refinement. Refinement of *F*^2^ against ALL reflections. The weighted *R*-factor *wR* and goodness of fit *S* are based on *F*^2^, conventional *R*-factors *R* are based on *F*, with *F* set to zero for negative *F*^2^. The threshold expression of *F*^2^ > σ(*F*^2^) is used only for calculating *R*-factors(gt) *etc*. and is not relevant to the choice of reflections for refinement. *R*-factors based on *F*^2^ are statistically about twice as large as those based on *F*, and *R*-factors based on ALL data will be even larger.

### Fractional atomic coordinates and isotropic or equivalent isotropic displacement parameters (Å^2^)

**Table:**

	*x*	*y*	*z*	*U*_iso_*/*U*_eq_	
Cl1	0.087385 (18)	0.57099 (12)	0.36030 (2)	0.02301 (15)	
O1	0.25626 (5)	0.5555 (3)	0.58943 (6)	0.0200 (3)	
N1	0.07663 (6)	0.3042 (4)	0.48893 (7)	0.0178 (3)	
N2	0.17290 (6)	0.5527 (4)	0.48745 (7)	0.0165 (3)	
C1	0.11297 (7)	0.4595 (4)	0.45572 (9)	0.0164 (3)	
C2	0.10115 (7)	0.2222 (4)	0.56609 (8)	0.0155 (3)	
C3	0.06280 (7)	0.0601 (4)	0.60639 (9)	0.0183 (3)	
H3	0.0211	0.0084	0.5814	0.022*	
C4	0.08592 (7)	−0.0240 (4)	0.68250 (9)	0.0186 (3)	
H4	0.0597	−0.1318	0.7098	0.022*	
C5	0.14730 (7)	0.0467 (4)	0.72020 (9)	0.0179 (3)	
H5	0.1626	−0.0140	0.7726	0.021*	
C6	0.18533 (7)	0.2042 (4)	0.68100 (9)	0.0172 (3)	
H6	0.2272	0.2508	0.7063	0.021*	
C7	0.16281 (6)	0.2959 (4)	0.60432 (8)	0.0144 (3)	
C8	0.20165 (7)	0.4738 (4)	0.56241 (9)	0.0166 (3)	
H1	0.1956 (9)	0.652 (6)	0.4567 (11)	0.042 (6)*	

### Atomic displacement parameters (Å^2^)

**Table:**

	*U*^11^	*U*^22^	*U*^33^	*U*^12^	*U*^13^	*U*^23^
Cl1	0.0253 (2)	0.0286 (3)	0.0141 (2)	−0.00270 (16)	0.00309 (15)	0.00145 (16)
O1	0.0127 (5)	0.0272 (6)	0.0195 (6)	−0.0031 (5)	0.0029 (4)	0.0031 (5)
N1	0.0165 (6)	0.0217 (7)	0.0150 (6)	−0.0028 (5)	0.0036 (5)	−0.0015 (6)
N2	0.0151 (6)	0.0201 (7)	0.0154 (6)	−0.0020 (5)	0.0061 (5)	−0.0002 (5)
C1	0.0171 (7)	0.0178 (8)	0.0136 (7)	0.0007 (6)	0.0028 (6)	−0.0023 (6)
C2	0.0166 (7)	0.0153 (8)	0.0147 (7)	0.0003 (6)	0.0040 (6)	−0.0022 (6)
C3	0.0152 (7)	0.0207 (8)	0.0196 (8)	−0.0032 (6)	0.0054 (6)	−0.0016 (7)
C4	0.0200 (8)	0.0203 (8)	0.0177 (8)	−0.0016 (6)	0.0087 (6)	−0.0003 (6)
C5	0.0220 (8)	0.0177 (8)	0.0141 (7)	0.0021 (6)	0.0047 (6)	0.0004 (6)
C6	0.0148 (7)	0.0178 (8)	0.0182 (7)	−0.0001 (6)	0.0028 (6)	−0.0020 (6)
C7	0.0145 (7)	0.0132 (8)	0.0161 (7)	0.0004 (6)	0.0049 (6)	−0.0021 (6)
C8	0.0174 (7)	0.0157 (8)	0.0176 (7)	0.0014 (6)	0.0060 (6)	−0.0010 (6)

### Geometric parameters (Å, º)

**Table:**

Cl1—C1	1.7249 (16)	C3—C4	1.378 (2)
O1—C8	1.2370 (18)	C3—H3	0.9500
N1—C1	1.2706 (19)	C4—C5	1.398 (2)
N1—C2	1.3971 (19)	C4—H4	0.9500
N2—C1	1.3669 (19)	C5—C6	1.374 (2)
N2—C8	1.3766 (19)	C5—H5	0.9500
N2—H1	0.92 (2)	C6—C7	1.392 (2)
C2—C3	1.400 (2)	C6—H6	0.9500
C2—C7	1.408 (2)	C7—C8	1.455 (2)
			
C1—N1—C2	115.79 (13)	C3—C4—H4	119.4
C1—N2—C8	121.52 (13)	C5—C4—H4	119.4
C1—N2—H1	119.1 (12)	C6—C5—C4	119.64 (14)
C8—N2—H1	119.2 (12)	C6—C5—H5	120.2
N1—C1—N2	126.93 (14)	C4—C5—H5	120.2
N1—C1—Cl1	119.55 (12)	C5—C6—C7	120.25 (14)
N2—C1—Cl1	113.53 (11)	C5—C6—H6	119.9
N1—C2—C3	118.46 (13)	C7—C6—H6	119.9
N1—C2—C7	122.36 (14)	C6—C7—C2	120.18 (14)
C3—C2—C7	119.18 (14)	C6—C7—C8	121.19 (13)
C4—C3—C2	119.58 (14)	C2—C7—C8	118.62 (14)
C4—C3—H3	120.2	O1—C8—N2	120.31 (14)
C2—C3—H3	120.2	O1—C8—C7	124.97 (14)
C3—C4—C5	121.16 (14)	N2—C8—C7	114.72 (13)
			
C2—N1—C1—N2	0.1 (2)	C5—C6—C7—C8	177.88 (14)
C2—N1—C1—Cl1	179.76 (11)	N1—C2—C7—C6	−179.03 (14)
C8—N2—C1—N1	1.8 (3)	C3—C2—C7—C6	0.8 (2)
C8—N2—C1—Cl1	−177.85 (11)	N1—C2—C7—C8	2.0 (2)
C1—N1—C2—C3	178.19 (13)	C3—C2—C7—C8	−178.21 (13)
C1—N1—C2—C7	−2.0 (2)	C1—N2—C8—O1	178.72 (13)
N1—C2—C3—C4	179.88 (14)	C1—N2—C8—C7	−1.7 (2)
C7—C2—C3—C4	0.1 (2)	C6—C7—C8—O1	0.5 (2)
C2—C3—C4—C5	−0.6 (2)	C2—C7—C8—O1	179.48 (15)
C3—C4—C5—C6	0.3 (2)	C6—C7—C8—N2	−179.07 (14)
C4—C5—C6—C7	0.5 (2)	C2—C7—C8—N2	−0.1 (2)
C5—C6—C7—C2	−1.1 (2)		

### Hydrogen-bond geometry (Å, º)

**Table:**

*D*—H···*A*	*D*—H	H···*A*	*D*···*A*	*D*—H···*A*
N2—H1···O1^i^	0.92 (2)	1.88 (2)	2.7840 (17)	166.5 (19)
C3—H3···N1^ii^	0.95	2.53	3.449 (2)	163

Symmetry codes: (i) −*x*+1/2, −*y*+3/2, −*z*+1; (ii) −*x*, −*y*, −*z*+1.
